# A Non-Contact Pulse Automatic Positioning Measurement System for Traditional Chinese Medicine

**DOI:** 10.3390/s150509899

**Published:** 2015-04-27

**Authors:** Ying-Yun Chen, Rong-Seng Chang, Ko-Wen Jwo, Chung-Chi Hsu, Chu-Pang Tsao

**Affiliations:** Department of Optics and Photonics of National Central University, No.300, Jhongda Rd, Taoyuan 32001, Taiwan; E-Mails: s102286007@dop.ncu.edu.tw (Y.-Y.C.); ko-wen@hotmail.com (K.-W.J.); aa8833.aa8833@yahoo.com.tw (C.-C.H.); tsaoukimo@yahoo.com.tw (C.-P.T.)

**Keywords:** non-contact, pulse, Traditional Chinese Medicine (TCM), optical triangulation measurements

## Abstract

This study is to construct a non-contact pulse automatic positioning measurement system for Traditional Chinese Medicine (TCM) using optical triangulation measurements. The system consists of a linear laser, a CMOS image sensor and image analysis software. The linear laser is projected on the pulse beat location on the wrists; the CMOS image sensor records the process and the software analyzes the images. The program mainly uses the optical centroid and fast Fourier transform (FFT) principles to calculate centroid changes (pulse amplitude changes) from the images taken by the CMOS image sensor. It returns the positions of cun, guan and chi pulses automatically in terms of the amplitudes and the signals are then transformed from the time domain (time-amplitude) into the frequency domain (frequency-amplitude) via FFT to obtain the waveforms and frequencies of the cun, guan and chi pulses. It successfully extracts the data from the TCM pulse reading and can be a medical aid system for TCM. Combining the advantages of optical measurement and computer automation, this system provides a non-contact, easy to operate, fast in detection and low-cost equipment design.

## 1. Introduction

**Figure 1:**
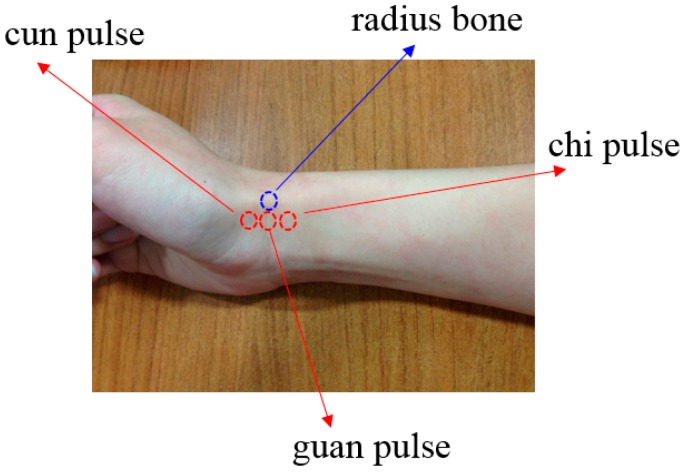
Cun, guan and chi pulse locations.

**Figure 2:**
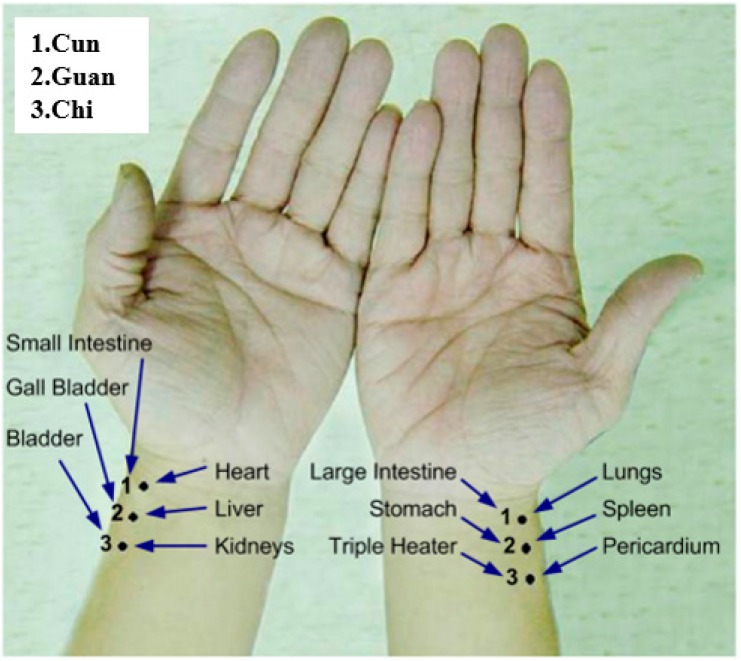
Correspondence between the three pulse points on both hands and the body organs.

Pulse reading in scientific terms is to capture the changes of the body’s pulse. According to TCM, the pulse is the blood fluctuation phenomenon triggered by the aortic wall’s elastic relaxation and contraction caused by cardiac ejection. The tip of the artery experiences a similar wave-like fluctuation, also known as the pulse wave [1,2,3,4]. Cardiac ejection can cause different states of blood volume, pressure and flow in arteries, creating different pulse beats that can be measured at the cun, guan and chi pulses which happen to be on the radial artery in Western medicine [5,6], as shown in Figure 1. The cardiovascular circulation is a closed fluid system and hence any problem with the system can impact the balance of the overall pulse system. We can use TCM pulse readings to learn about the body's health. Figure 2 shows the cun, guan and chi pulse locations, corresponding to the functioning of two organs [7] (pp. 122–130; pp. 239–243).

This study aims at constructing a non-contact optical pulse measuring instrument mainly consisting of a linear laser, a CCD image capturing sensor and image analysis software. The linear laser beam is projected on the radial artery with the returned amplitudes that automatically position the cun, guan and chi locations which are then translated into the waveforms and frequency [8,9,10]. Without instrumental analysis, the doctors currently practice TCM using their fingers by applying varying pressures to read pulses according to their own experiences and sensations and therefore, the TCM practice can only express the pulse reading by words, but it cannot be quantified. In science, the description needs to be qualitative as well as quantitative. A quantified pulse reading can better be compared, as every doctor in the traditional practice feels differently and it takes great effort to confirm the descriptions [11,12]. Taking advantage of the wave nature of the pulses, this system organizes the pulse reading in TCM into preliminary data, further helping the pulse diagnosis.

Some current pulse measuring equipment uses the piezoelectric method [13], and the disadvantages are as follows: first, the sensor pressures the pulse location. There exist changes in the tension of skin soft tissues and the artery’s axial tension, and the vascular radial pulse also exerts force upon contact. In terms of measuring the radial pulse, the impact of this tension cannot be eliminated, and therefore the pulse related to vascular tension and the pulse related to radial direction are indistinguishable in their characteristics [14,15,16]. Second, the sensor pressures the skin to read pulse, and due to the pressure feedback (pulse pressure) from the skin soft tissue the pressured contact may experience deformations, causing bigger measurement errors [17,18].

## 2. Research Method and System Configuration

This system applies an optical triangulation method to form laser spots which are later translated into laser lines through a cylindrical lens. A linear laser beam is projected on the wrist where the pulse vibration is, and a CMOS image sensor records the light variations on the wrist. Using a thresholding technique, the software converts all images taken into binary images and the variation of centroid position (amplitude variation) can be achieved. The position of the cun, guan and chi pulses can be automatically located at points where the maximum amplitude variation is observed. Taking the time domain waveform for cun, guan and chi pulses into fast Fourier transform (FFT) the frequency domain data is returned. The following sections illustrate the principle involved.

### 2.1. The Principle of Optical Triangulation Measurement

**Figure 3:**
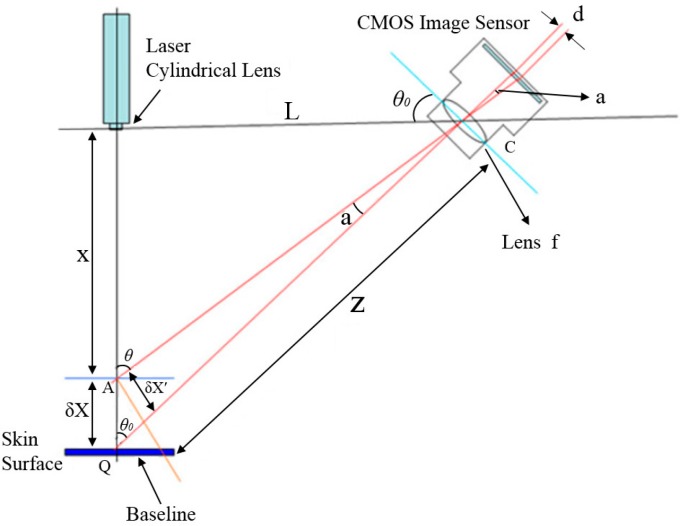
Schematic showing optical triangulation measurement.

Triangulation measurements form a triangle structure between the light source, the subject to be tested and detectors to calculate the amplitude change on the surface of the test subject. As shown in Figure 3, the light source in this system is a red laser, the test subject is the pulse on the skin surface of the wrist and the detector is a CMOS image sensor.

where:

X: The distance between the laser collimator lens and the tested object.
δX: Amplitude of the wrist pulse vibration.
*L*: The distance between laser and the CMOS image sensor.
*d*: The distance between the two bright spots projected on the CMOS image sensor.
*f*: Focal length of the lens.
Z: The distance between the center point C of the lens in the CMOS image sensor and the baseline.
a: The angle between the tested spot A and the central axis of the lens in the CMOS image sensor.
$\text{δ}X^{\prime }$: The distance of central axis between the tested spot A and the central axis of the lens in the CMOS image sensor.
*θ*_0_: The angle between laser at the baseline and the CMOS image sensor.

Using a simple triangulation principle, the target coordinates X are mapped to the detection position d on the sensor. X is

(1) $$\text{X}=\frac{L}{\tan\left[\theta_{0}+\tan^{-1}\left(\frac{d}{f}\right)\right]}$$

We measure the displacement *δX* on the skin surface resulting from pulses, and both by the simple triangulation method and by dfferentiating Equation (1) with respect to X, we can get δX, as the result of [8,9]:

(2) $$\text{δX}=\frac{Z^{2}d}{fL}$$

**Figure 4:**
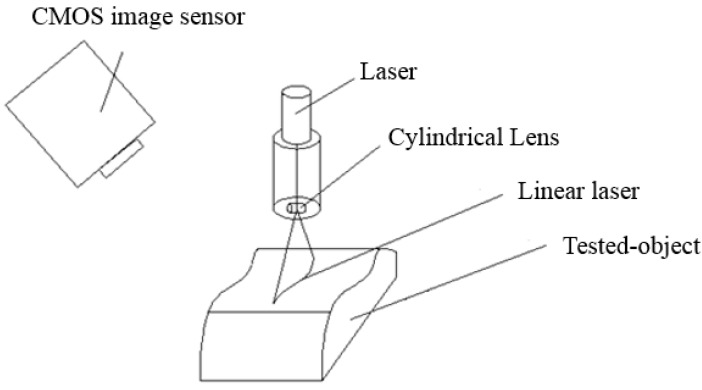
Projecting a linear laser on the tested object.

This study uses a cylindrical lens to convert the laser spot into a linear laser beam, projecting the laser on the object to record the surface change of the laser projection as shown in Figure 4.

### 2.2. Optical Centroid

**Figure 5:**
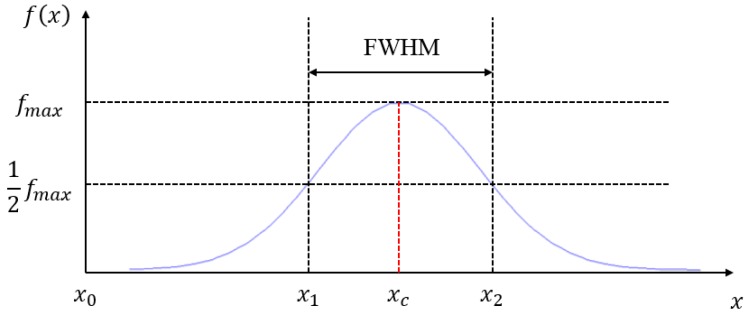
Laser intensity function and the centroid position.

We process and analyze the images that record the deformation of the laser beam and divide the linear laser beam into several spots in units of pixels. Then we calculate the centroid position of the laser spots in the drawing and use Equation (2) in the optical triangulation measurement to convert the pixel location of each centroid into the actual height position related to each point. The calculation of the optical centroid is performed as follows: the laser intensity detected by the CMOS image sensor can be approximated to a Gaussian distribution. The centroid position is defined as the center of the two sides of the full width at half maximum (FWHM) of the laser intensity, as shown in Figure 5.

According to Figure 5, centroid position can be written as:

(3) $$x_{c}=\frac{x_{2}-x_{1}}{2}+x_{0}$$

where *x_c_* is the centroid position and *x*_0_ is the reference point (this study sets the radius bone as the reference point).

Therefore, through linear laser triangulation and optical centroid calculation, we can obtain the change (time-amplitude) of the signal amplitude on the object surface. To make the data more meaningful, we use a Fast Fourier Transform (FFT) to convert it to “frequency–amplitude” and convert the time domain into the frequency domain to allow us obtain more information about the object surface.

### 2.3. System Architecture

**Figure 6:**
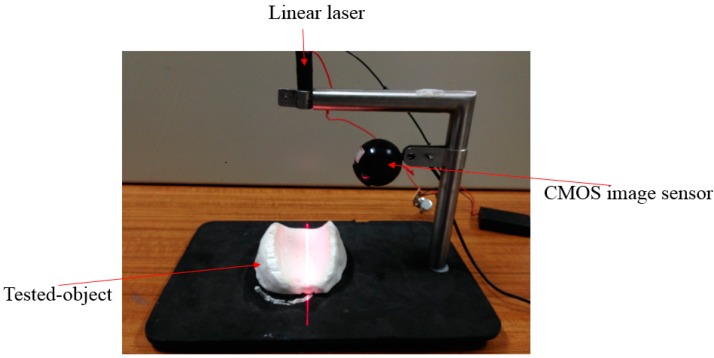
Non-contact pulse automatic positioning measurement system for TCM.

The system is built based on the abovementioned principle. The system consists of a linear laser, CMOS image sensor and an object to be measured, as shown in Figure 6. The captured image is then analyzed by the self-developed program.

**Figure 7:**
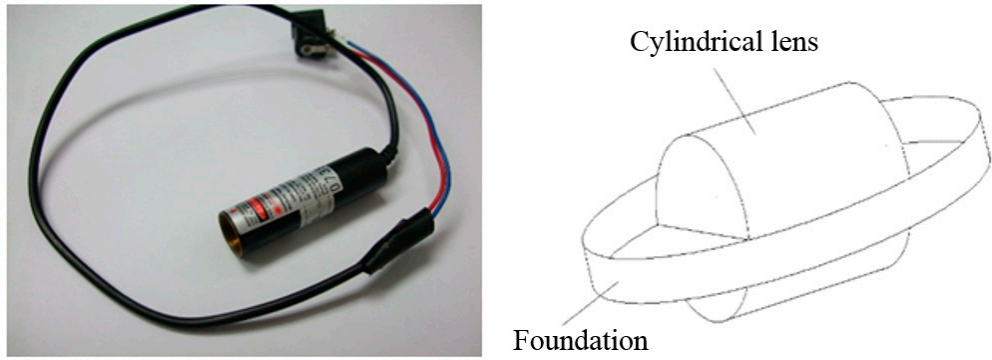
Red line Laser Diode Module and a schematic showing the cylindrical lens.

The linear laser used in this study is a Laser Diode Module. Since the beam divergence angle is quite wide, around 35° × 11°, and is distributed in an oval shape, this study uses these properties to place its light-emitting area on the focus of a cylindrical lens, calibrating one of the directions (the direction of the minor axis with lens flare or of small divergence angle). In the other direction (the direction of the long axis or of large divergence angle), it is freely divergent, projecting the laser beam in space. Its wavelength ranges from 635 nm to 680 nm and the output power of the device is 3 mW. There is a zoom lens in the front. The red line Laser Diode Modules and a cylindrical lens are shown in Figure 7. The resolution for the CMOS image sensor is 640 × 480 pixels and the shooting speed is 30 frames/s.

### 2.4. Experimental Process

**Figure 8:**
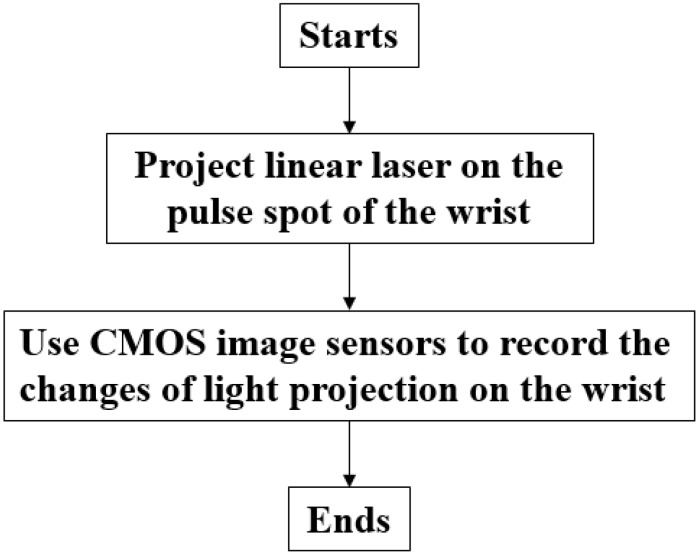
The process for non-contact pulse automatic positioning measurement system for TCM.

**Figure 9:**
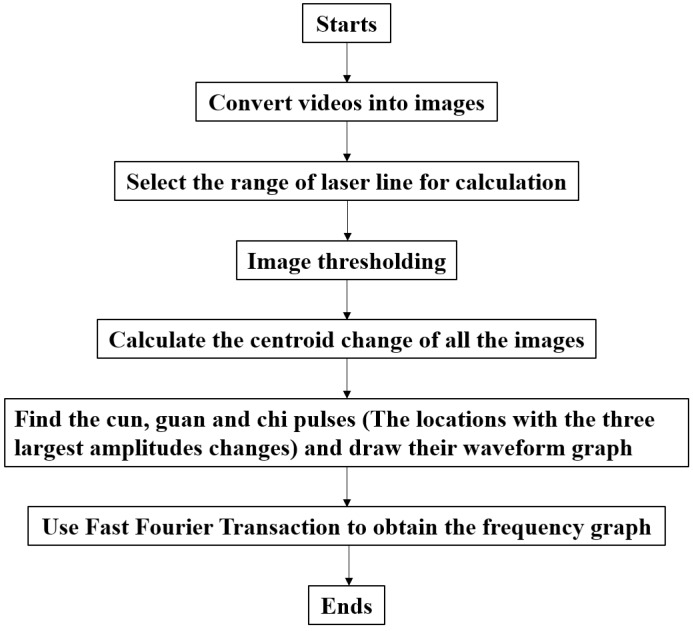
Program flow chart.

The process for the non-contact pulse automatic positioning measurement system for TCM is shown in Figure 8. It first aims a linear laser beam at the pulse location on the wrist, and then records for 10 s. The video is then sent to a computer for analysis. The program flow chart is shown in Figure 9. The video is divided into 30 (frames) × 10 (s), for a total of 300 images. As the light source being used in this system is red light, it is necessary to resolve the R value from the image containing R, G, and B color components before the thresholding technique is applied.

**Figure 10:**
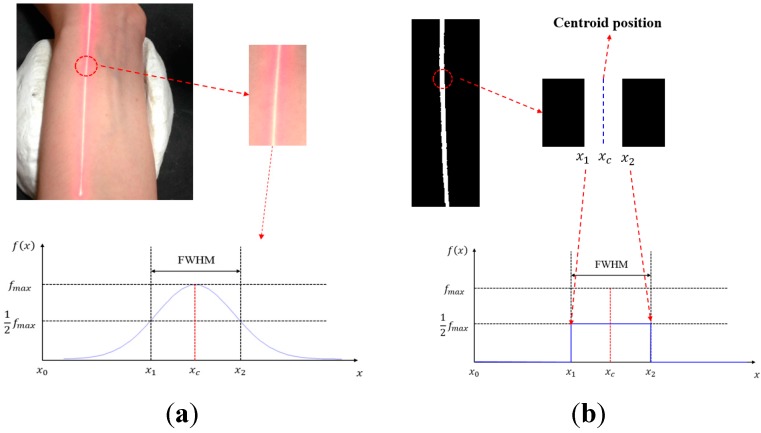
The image and distribution of laser intensity. (**a**) before thresholding; (**b**) after thresholding.

The threshold value is calculated by FWHM of the laser intensity ($\frac{1}{2}f_{\max}$). For intensities larger than the threshold value, such an intensity is applied as ($\frac{1}{2}f_{\max}$); for intensities lower than the threshold value, it returns 0, as shown in Equation (4) and Figure 10:

(4) $$\{\begin{array}{c} if\;f\left(x\right)\geq \frac{1}{2}f_{\max}\;,\;\mathrm{then}\;f\left(x\right)=\frac{1}{2}f_{\max}\;\\ f\left(x\right)<\frac{1}{2}f_{\max}\;,\;\mathrm{then}\;f\left(x\right)=0\; \end{array}$$

Since the background environment remains unchanged during the measurement, the threshold value is fixed. The optical centroid method is used to calculate the centroid position of the laser, obtaining the amplitude of the linear laser beam of every image. The locations with the three largest amplitudes changes, representing cun, guan and chi, are used to analyze their pulse waveforms and frequency graphs.

### 2.5. Height Displacement Calibration

Since this system analyzes the centroid position of laser spots on the test subject by the images captured by the CMOS image sensor in pixel units, we need to perform a height displacement calibration to calculate the amount of distance change per pixel movement of the laser central position.

**Figure 11:**
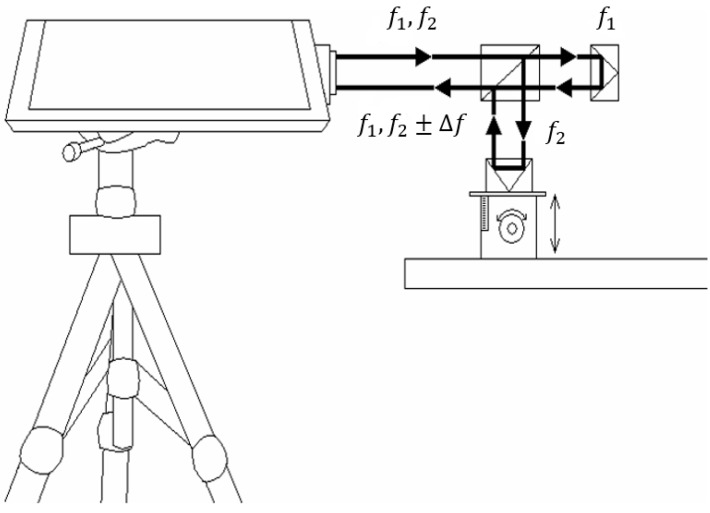
A schematic of a laser interferometer.

This study uses a laser range finder to calibrate the actual height displacement. The range finder is a laser interferometer manufactured by Agilent Technologies (Santa Clara, CA, USA). Figure 11 shows the schematic of a laser interferometer. The laser head has two holes, emitting dual frequency laser beams (f_1_ and f_2_) out from the top and receiving signals at the bottom. A polarizing beam splitter divides the incoming laser into two beams at frequency f_1_ and f_2_, where the f_1_ beam is used as the reference frequency. After phase shifting and other physical processes, the reflected f_2_ beam which experienced a phase change (f_2_ ± △f) and the reflected f_1_ beam at its original frequency are both received by the laser head and generate the composite wave of beat frequency (f_1_ and f_2_ ± △f). A built-in interface card then interprets the frequency and obtains the measurement results.

**Figure 12:**
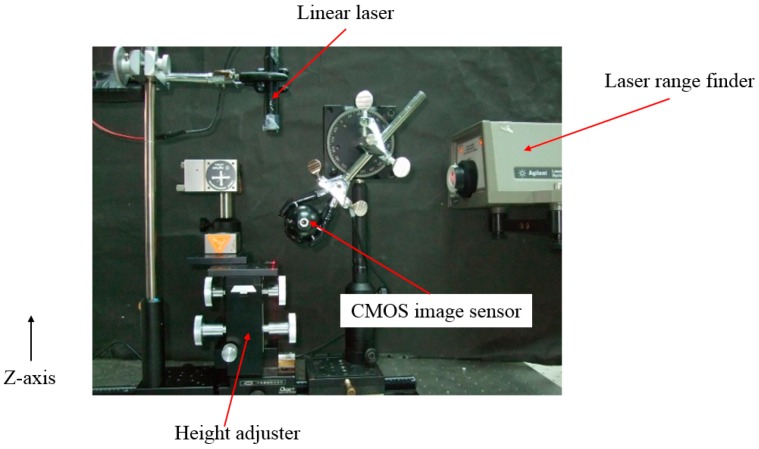
Use of a laser range finder to synchronize the height adjuster.

**Figure 13:**
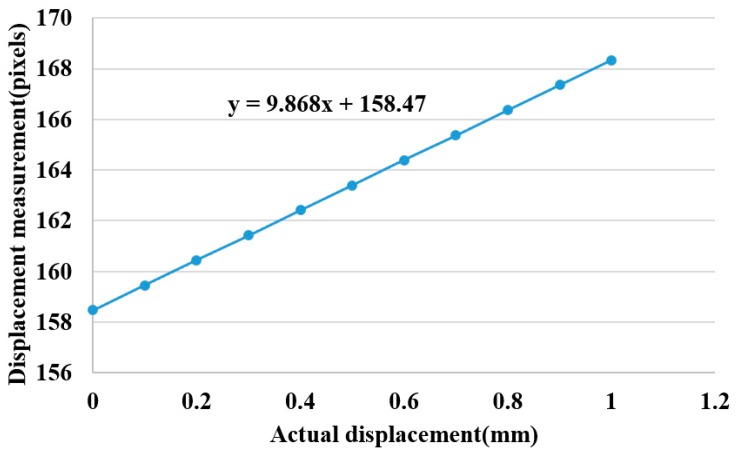
Height displacement calibration.

A laser range finder is used to calibrate the height change of a height adjuster. The laser centroid position is recorded for a movement of 0.1 ± 0.01 mm on the *Z*-axis. The total movement of 10 repetitions is 1 mm, as shown in Figure 12 where the laser range finder calibrates the height adjustments on the fly, producing an error under 0.01 mm. The calibration results are shown in Figure 13. The results show a linear correlation between the measured displacement and the actual displacement and a trend line given by the expression *y* = 9.868*x* + 158.47. From this we conclude that the centroid position moves 101 μm for every 1 pixel of movement detected.

### 2.6. The System of Frequency Precision Measurement

**Figure 14:**
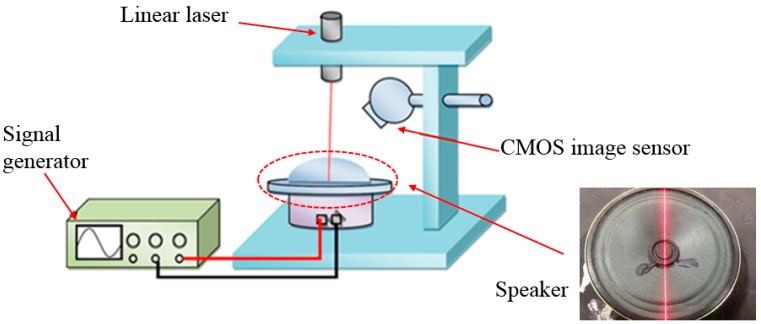
The experimental architecture of the frequency precision measurements.

In order to verify the precision of this system before performing actual pulse measurements, we connect a speaker to the signal generator which is monitored by an oscilloscope to produce a stable frequency that is accurate to the second decimal place. We adjust the frequency from 0.60 Hz to 2.00 Hz in increments of 0.10 Hz. The system measures the precision of each frequency with the actual configuration shown in Figure 14. The result of frequency precision measurements returns an error within 2.5%, as shown in Table 1.

**Table 1:** Results of frequency precision measurement.

Input Frequency (Hz)	Measured Frequency (Hz)	Errors (%)
0.60	0.59	1.67%
0.70	0.69	1.42%
0.80	0.81	1.25%
0.90	0.89	1.11%
1.00	0.98	2.00%
1.10	1.11	0.90%
1.20	1.18	1.67%
1.30	1.32	1.54%
1.40	1.38	1.43%
1.50	1.51	0.67%
1.60	1.58	1.25%
1.70	1.68	1.18%
1.80	1.81	0.56%
1.90	1.89	0.52%
2.00	2.00	0.00%

## 3. Process and Pulse Measurement Results

**Figure 15:**
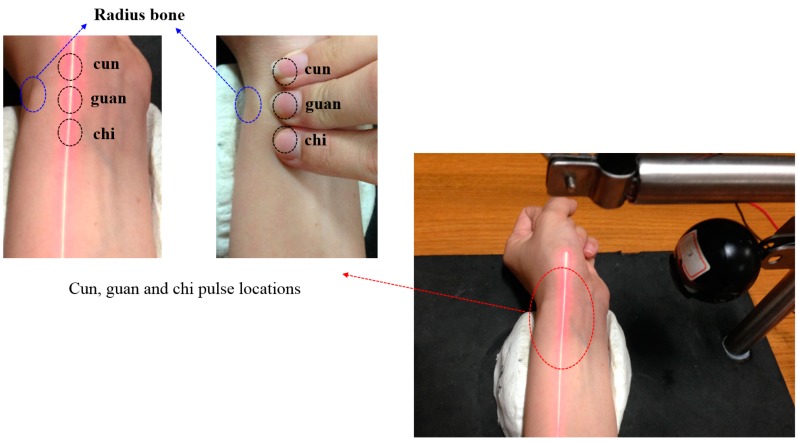
Measurement process.

**Figure 16:**
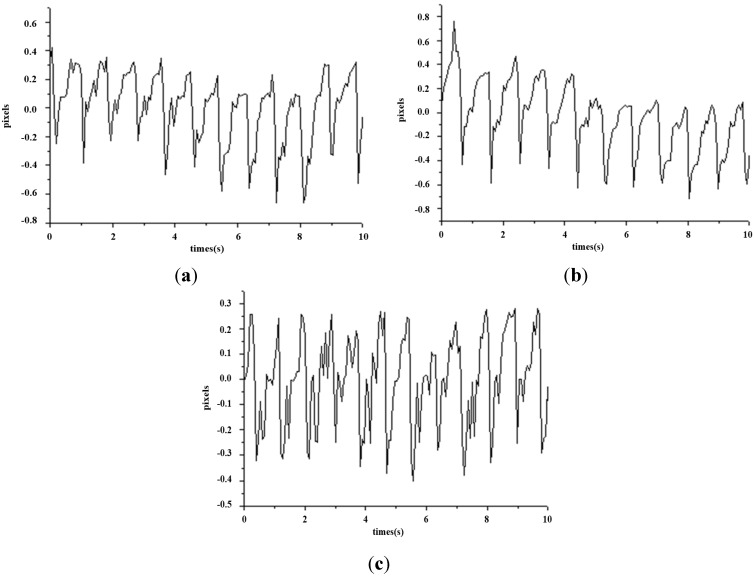
Waveforms for the cun, guan and chi pulses (time–amplitude). (**a**) Waveform for cun pulse (time–amplitude); (**b**) Waveform for guan pulse (time–amplitude); (**c**) Waveform for chi pulse (time–amplitude).

**Figure 17:**
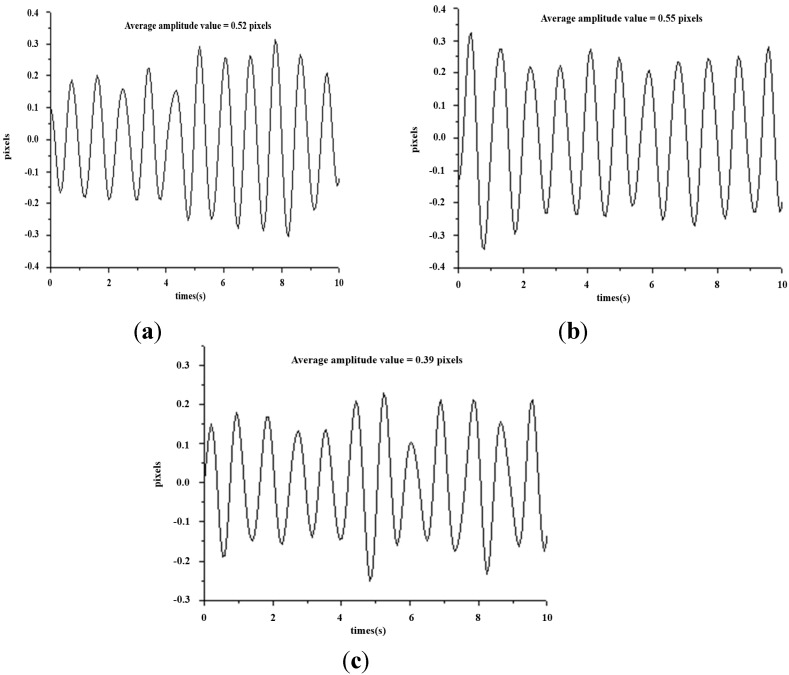
Band-pass filtering graph for the cun, guan and chi pulses. (**a**) Band-pass filtering graph for cun pulse; (**b**) Band-pass filtering graph for guan pulse; (**c**) Band-pass filtering graph for chi pulse.

**Figure 18:**
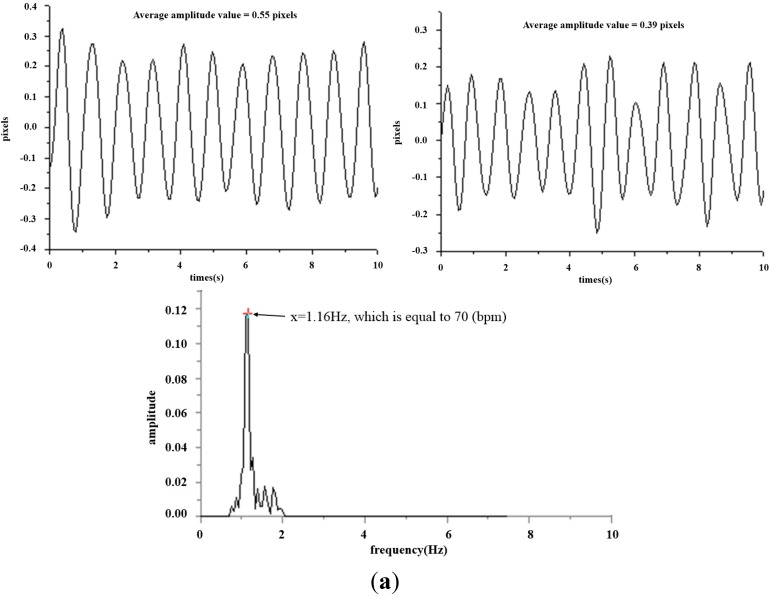

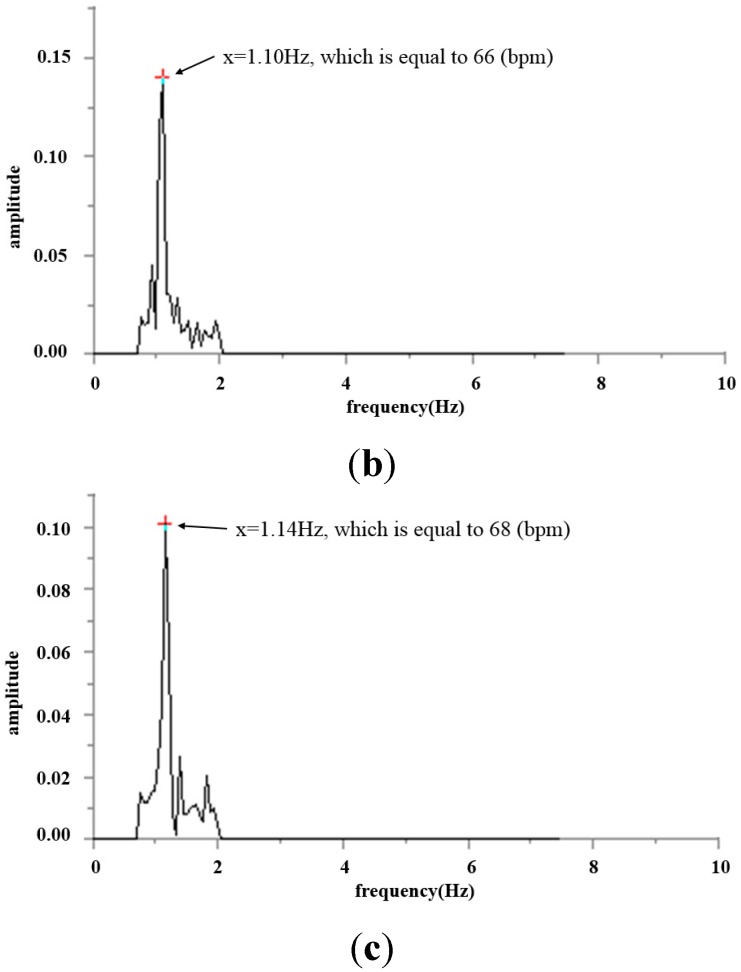
Heart beat frequency graph (frequency–amplitude) for cun, guan and chi pulses. (**a**) Heartbeat frequency graph (frequency–amplitude) for the cun pulse; (**b**) Heart beat frequency graph (frequency–amplitude) for guan pulse; (**c**) Heart beat frequency graph (frequency–amplitude) for chi pulse.

This study mainly applies the concept of TCM pulse reading to pulse measurement using optical triangulation. To maintain a consistent laser projection on the cun, guan and chi pulses, this system proposes a two-step aiming method: we first find the radius bone position, and then we use it as a reference in search of pulses. This originates from the description listed in Traditional Chinese Medicine, “The part slightly below the styloid process of radius bone is the guan pulse; the part anterior the guan pulse is the cun pulse, and the part posterior the guan pulse is the chi pulse [7].” Hence, we use the coordinates of the image to locate the positions of the cun, guan and chi pulses. For each measurement, we use a traditional Chinese physician’s finger to reconfirm the position of cun, guan and chi pulses, as shown in Figure 15. The actual images are captured and then analyzed by software. The locations with the three largest amplitudes changes, representing the cun, guan and chi pulses, are found and the waveforms are analyzed in the time domain (time–amplitude) which are later converted to the frequency domain (frequency–amplitude), or heartbeat rate (BPM), using a Fast Fourier Transform (FFT). As the pulse of a normal person ranges from 60 to 100 beats per minute, we set the band-pass filter at 0.7 Hz to 2.0 Hz. The program filters out signals with a frequency less than 0.7 Hz or larger than 2.0 Hz and records the main frequency and amplitude. Figure 16 shows the waveforms of different pulses detected at the cun, guan and chi locations. Figure 17 is the band-pass filtering process for the three pulses. Figure 18 is the frequency graph showing the three pulses (frequency-amplitude).

Figure 16 shows the waveform of the three pulses which are unique from one another. This reflects the uniqueness of every organ in the participants, as indicated in Figure 2. Figure 17 is the band-pass filtered graph for the three pulses where the average amplitude is calculated. According to Section 2.5, the calibrated results obtained from the height displacement experiment show that the centroid position moves 101 μm for every 1 pixel movement detected. This returns the actual height displacement of the three pulses. Finally, via Fast Fourier Transform (FFT), a heart beats frequency graph (Figure 18) is achieved. The difference in the heart beat frequency between the three pulses is far from obvious and hence is not ideal for determining physiological signs. Therefore, to determine physiological signs, waveform and actual height of pulses are used.

The results from Table 2 demonstrate the current condition of the pulses with respect to the sensation of pulse felt by the finger, in accordance with Chinese traditional medicine principles. The purpose of the examination of pulse conditions is to distinguish the features of two different pulse features–the rhythm of the pulse and the strength of the pulse. Currently pulse meters available on market can only measure the rhythm of the pulse while this research measures both the rhythm of pulse via frequency and the strength of pulse via average amplitude. We quantified the pulse strength in terms of actual height variation. This makes the conventional method which determines the strength of pulse through the sensations felt by a traditional Chinese physician’s finger no longer needed and is the innovative contribution of this study.

**Table 2:** Average amplitude (μm) corresponding to the main frequencies of cun, guan and chi pulses.

	Cun	Guan	Chi
Average Amplitude (μm)	52.52 (μm)	55.55 (μm)	39.39 (μm)

To show the reliability of this system, we used a commercially available wrist pulse instrument (model number: Prince-100G, Heal Force, Shanghai, China) and compared it to this system. As the commercially available pulse instruments cannot distinguish the heartbeat rate (frequency, in bpm) between the cun, guan and chi pulses, we compare the heartbeat rates (frequency) by taking the average out of the three rates measured by this system. Ten testers were involved, and the heart rates (frequency) are shown in Table 3, all with error less than 5%.

**Table 3:** Results of measurement.

Number of the Tested Person	Prince-100G (bpm)	Proposed System (bpm)	Error (%)
1	80	82	2.50
2	75	74	1.33
3	77	80	3.90
4	92	89	3.26
5	65	67	3.08
6	74	72	2.70
7	85	83	2.35
8	96	93	3.13
9	69	72	4.35
10	70	69	1.43

## 4. Conclusions

The multiple measurement results presented in this study have proved the feasibility of the proposed system in terms of its application in pulse measurement using the above-mentioned pulse to amplitude change monitoring technique. Exploiting the pulse waveforms and frequency graphs as well as TCM theories, the measured cun, guan and chi pulses reflect changes of human physiology. Thus, this system can aid physicians to diagnose the pulse condition, and it can also serve as a simple pulse measurement for individuals. In other words, this system can be viewed as a prototype of an optical pulse meter.

In the future, we will cooperate with other conventional medicine-related organizations to calibrate the system aiming at enhancing the performance. According to TCM theory, we may explore other physiological status aspects and their corresponding pulse positions or conditions. Last but not least, running trials on subjects and collecting data, are essential to make the results of this system to more relevant to the requirements of TCM.
